# LncRNA PVT1 regulates CD4 + T cell dysregulation in systemic lupus erythematosus: insights from human patients and MRL/lpr mouse

**DOI:** 10.1007/s10067-025-07519-4

**Published:** 2025-06-10

**Authors:** Jiali Zhang, Ying Yuan, Shuangying Ni, Siqi Mu, Wanrong Wang, Feiyang Sun, Bo Liang, Peng Lu, Yue Qiu, Wenhui Du, Chenjun Wang, Huijie Duan, Zejuan Hu, Leilei Wen, Xiaodong Zheng, Yujun Sheng, Shengquan Zhang, Shanyu Chen, Xueli Yin, Zhengwei Zhu

**Affiliations:** 1 Institute of Dermatology and Department of Dermatology, the First Affiliated Hospital, Anhui Medical University, Hefei, China; 2 Key Laboratory of Dermatology (Anhui Medical University), Ministry of Education, Hefei, China; 3 Functional Experiment Center, School of Basic Medical Sciences, Anhui Medical University, Hefei, China; 4 Department of Rheumatology and Immunology, The First Affiliated Hospital of Anhui Medical University, Hefei, China; 5 Department of Dermatology, Shannan People’s Hospital, Shannan, China; 6 Department of Biochemistry and Molecular Biology, School of Basic Medical Sciences, Anhui Medical University, Hefei, China; 7 First Clinical Medical College, Anhui Medical University, Hefei, China; 8 Department of Dermatology, Friendship Hospital, Beijing, China

**Keywords:** LncRNA PVT1, Systemic lupus erythematosus (SLE), CD4^+^T cells, MRL/lpr mice, MiRNA-30e-5p

## Abstract

**Objectives:**

To investigate the role of lncRNA PVT1 in modulating CD4^+^ T cell subsets and its contribution to systemic lupus erythematosus (SLE) pathogenesis in human patients and MRL/lpr mice.

**Methods:**

Measured PVT1 and miR-30e-5p expression in SLE patients (*n* = 65) and healthy controls (HCs) using qRT-PCR. Analyzed Th1/Th2/Th17/Treg cell frequencies by flow cytometry and cytokine levels (IL-2, IL-4, IL-6, IL-17, TGF-β) via ELISA. Constructed lentiviral vectors to silence (SLE + si-*Pvt1*) or overexpress *Pvt1* (SLE + lenti-*Pvt1*) in MRL/lpr mice (*n* = 40).

**Results:**

PVT1 was upregulated (*p* = 0.0488) and miR-30e-5p downregulated (*p* = 0.0095) in SLE patients. Th2 (*p* = 0.0165) and Th17 (*p* = 0.0017) cells exhibited a significant increase, while Th1 and Treg cells decreased. *Pvt1* silencing reversed SLE phenotypes, increasing Th1 and Treg cells, reducing Th2 and Th17 cells, restoring IL-2 and TGF-β levels and reducing levels of IL-6 and IL-17. Overexpression of *Pvt1* exacerbated disease severity. *Pvt1* acted as a ceRNA to sponge miR-30e-5p, modulating T-bet/GATA3/RORγt/Foxp3 expression.

**Conclusions:**

PVT1 dysregulation disrupts CD4^+^ T cell homeostasis in SLE. Targeting the PVT1/miR-30e-5p axis may restore immune balance and represent a novel therapeutic strategy.

**Table:** 

*Key Points* • *Our data confirm the imbalance of CD4+ T cell subsets in SLE patients and demonstrate specific upregulation of lncRNA PVT1 expression in female SLE patients.* • *Targeting lncRNA PVT1 affects Th1/Th2 and Th17/Treg homeostasis in MRL/lpr mice.* • *Offers fresh insight into the dysregulation of lymphocyte subsets in SLE.*

## Introduction

Systemic lupus erythematosus (SLE) is a prototypical autoimmune disease that may cause chronic inflammation and debilitating damage in multiple organs and tissues [1]. It is characterized by a global breakdown of immune tolerance with activation of both innate and adaptive immune responses against nucleic acids and endogenous antigens [2]. Though still with an unclear disease of origin, the chance of developing SLE is believed to be associated with genetic factors, epigenetic factors, environmental triggers, and hormonal factors [3].

There are two types of T cells, i.e., αβ^+^ and γδ^+^ T cells, and αβ^+^ T cells can be further classified into CD4 + and CD8 + T cells. While CD8 + T cells take on the cell-killing activity [4], CD4 + T cells are T helper (Th) cells capable of promoting CD8 + T cell development and B cell differentiation as well as antibody synthesis [5] [6]. CD4 + T cells can be roughly subdivided into Th1, Th2, Th17, and regulatory T (Treg) cells, based on their distinct cytokine profilings [7]. Cytokines produced by Th1 cells are largely pro-inflammatory, and those generated by Th2 cells are primarily anti-inflammatory [8]. Numerous pieces of evidence have suggested that abnormal T cell differentiation to Th2 dominance can lead to B cell hyperactivation that contributes to immune disorders including SLE pathogenesis [9]. While IL12 drives Th1 cell differentiation through STAT4 signaling that leads to upregulated IFN-γ and downregulated IL4/IL5 for amplified Th1 proliferation, IL4 induces Th2 clonal expansion through STAT6 that results in upregulated levels of IL4/IL5 and downregulated IFN-γ expression for augmented Th2 differentiation [10]. The imbalance between pro-inflammatory Th17 cells and immuno-suppressive Tregs underlies the pathogenesis of SLE [11] [12]. The proportion of Th17 cells is higher in SLE patients, the content of which is positively correlated with SLE severity [13]. Tregs play an important role in maintaining immune tolerance, reduced levels or activities of which are tightly associated with the onset and progression of SLE [14]. The imbalance of CD4 + T cell subsets in SLE is the core pathological mechanism, but how to accurately regulate its differentiation network to restore immune homeostasis remains a scientific and clinical problem that needs to be solved urgently. While cytokines and transcription factors (e.g., T-bet, GATA3, RORγt, Foxp3) are known to regulate this imbalance, emerging evidence highlights the critical role of epigenetic regulators, particularly long non-coding RNAs (lncRNAs), in shaping CD4 + T cell fate [15].

Epigenetic mechanisms, such as DNA methylation, chromatin remodeling, and non-coding RNAs (ncRNAs), have been identified as key regulatory factors in immune responses [16]. Long non-coding RNAs (lncRNAs) are RNA transcripts that exceed 200 base pairs in length and lack protein-coding capacity, but they function as regulatory molecules influencing numerous biological processes in cells and organs [17]. Extensive research has revealed aberrant ncRNA patterns and their potential involvement in the pathogenesis of SLE [18]. The plasmacytoma variant translocation 1 (PVT1), located at the well-known genomic locus upstream of the c-Myc oncogene (8q24), is associated with Bcl2-mediated apoptosis [19]. Aberrant expression of lncRNAs, including FAS-AS1 and PVT1, has been observed in autoimmune diseases such as rheumatoid arthritis [20], multiple sclerosis [21], and Sjögren’s syndrome [22]. Recent studies have suggested that circular noncellular components of lncRNAs can serve as noninvasive biomarkers for diseases like SLE [23]. Fu et al. [24] demonstrated that the upregulation of lncRNA PVT1 in CD4 + T cells from patients with Sjögren’s syndrome can sustain Myc expression, thereby controlling the proliferation and effector function of CD4 + T cells through the regulation of glycolytic reprogramming. However, its mechanistic contribution to SLE-associated CD4 + T cell dysregulation remains unexplored, representing a critical knowledge gap.

Despite extensive research into the relationship between coding regions and single-nucleotide polymorphisms in SLE pathogenesis, further investigations are needed to establish the significance of non-coding regions as critical risk factors for SLE. Current studies have largely focused on individual cytokines; however, a greater emphasis on the synergistic effects of multiple cytokines is necessary for a comprehensive understanding. Additionally, more research is required to explore the extracellular factors that shape SLE T cells within the lupus environment. Preliminary findings from our research group suggest an interaction between miR-30e-5p and lncRNA PVT1. This study aims to investigate the potential impact of the lncRNA PVT1/miR-30e-5p axis on the proliferation and activation of distinct CD4^+^ T cell subpopulations, as well as its influence on the occurrence and progression of SLE and the specific regulatory relationships involved.

## Materials and methods

### Subjects

This study included a total of 65 fresh peripheral blood samples obtained from SLE patients who were hospitalized in the Department of Rheumatology and Department of Dermatology at the First Affiliated Hospital of Anhui Medical University between January 2020 and December 2022. The patients met the SLE classification and diagnostic criteria of the American College of Rheumatology (ACR), as revised in 1997 [25]. All enrolled patients were women between the ages of 18 and 50, and they were excluded if they had co-infections, tumors, or other autoimmune diseases. Blood samples were collected on the second day of admission, and they had not received hormone therapy for at least 3 months. Sixty-five age-matched health controls (HCs) were included in the study. Their fresh peripheral blood samples were obtained from the Health Examination Center of the First Affiliated Hospital of Anhui Medical University. They had no history of rheumatoid immunity or family history of rheumatoid immunity diseases. Informed consent forms were signed by all enrolled SLE patients and HCs. Patients meeting any of the following conditions were excluded from the study: uncontrolled infections such as pneumonia (bacterial, viral, or fungal), pulmonary tuberculosis, hepatitis B and C, skin infection, or central nervous system infection; persistent or unresolved inflammation including atherosclerosis, chronic obstructive pulmonary disease, diabetes, rheumatoid arthritis, Alzheimer’s disease, or amyotrophic lateral sclerosis; patients who had taken aspirin or fish oil within the past month; individuals with a history of cancer within the past 5 years: pregnant or lactating women. The Biomedical Ethics Committee of Anhui Medical University approved this study (for detailed experimental methods and materials, please see the Supplementary Material).

## Results

### Demographic and clinical characteristics of the patients studied

This study enrolled 65 female patients with active SLE (mean age 42.78 ± 13.96 years) and 65 age- and sex-matched healthy controls (HCs). Key clinical features of SLE patients included hypocomplementemia (70.77%), elevated anti-dsDNA antibody levels (55.38%), and arthritis (50.77%). The mean SLEDAI-2 K score was 11.77 ± 5.24, with a disease duration of 7.73 ± 6.72 years, indicating moderate-to-severe disease activity and chronic progression. Detailed demographic and clinical parameters are summarized in Table 1.

**Table 1:** Demographic and clinical characteristics of the SLE patients (*n* = 65)

Parameters	SLE patients (*n* = 65)
Gender: female/male	65/0
Age (mean ± SD)	42.78 ± 13.96
Disease duration (years) (mean ± SD)	7.73 ± 6.72
Seizure	0.00%
Psychosis	0.00%
Organic brain Syndrome	0.00%
Visual disturbance	1.54%
Cranial nerve disorder	0.00%
Lupus headache	3.08%
CVA	0.00%
Vasculitis	0.00%
Arthritis	50.77%
Myositis	6.15%
Urinary casts	1.54%
Hematuria	35.38%
Proteinuria	53.85%
Pyuria	27.69%
New rash	27.69%
Alopecia	16.92%
Mucosal ulcers	1.54%
Pleurisy	3.08%
Pericarditis	1.54%
Low complement	70.77%
Increased DNA binding	55.38%
Fever	20.00%
Thrombocytopenia	26.15%
Leukopenia	26.15%
SLEDAI-2 K (mean ± SD)	11.77 ± 5.24

*SD*, standard deviation; *SLEDAI-2 K*, Systemic Lupus Erythematosus Disease Activity Index 2000

### Increased lncRNA PVT1 in SLE patients

Fresh peripheral blood was collected in 5-mL purple-top anticoagulant tubes and processed within 4 h to ensure viability of CD4 + T cell subsets.

**Fig. 1:**
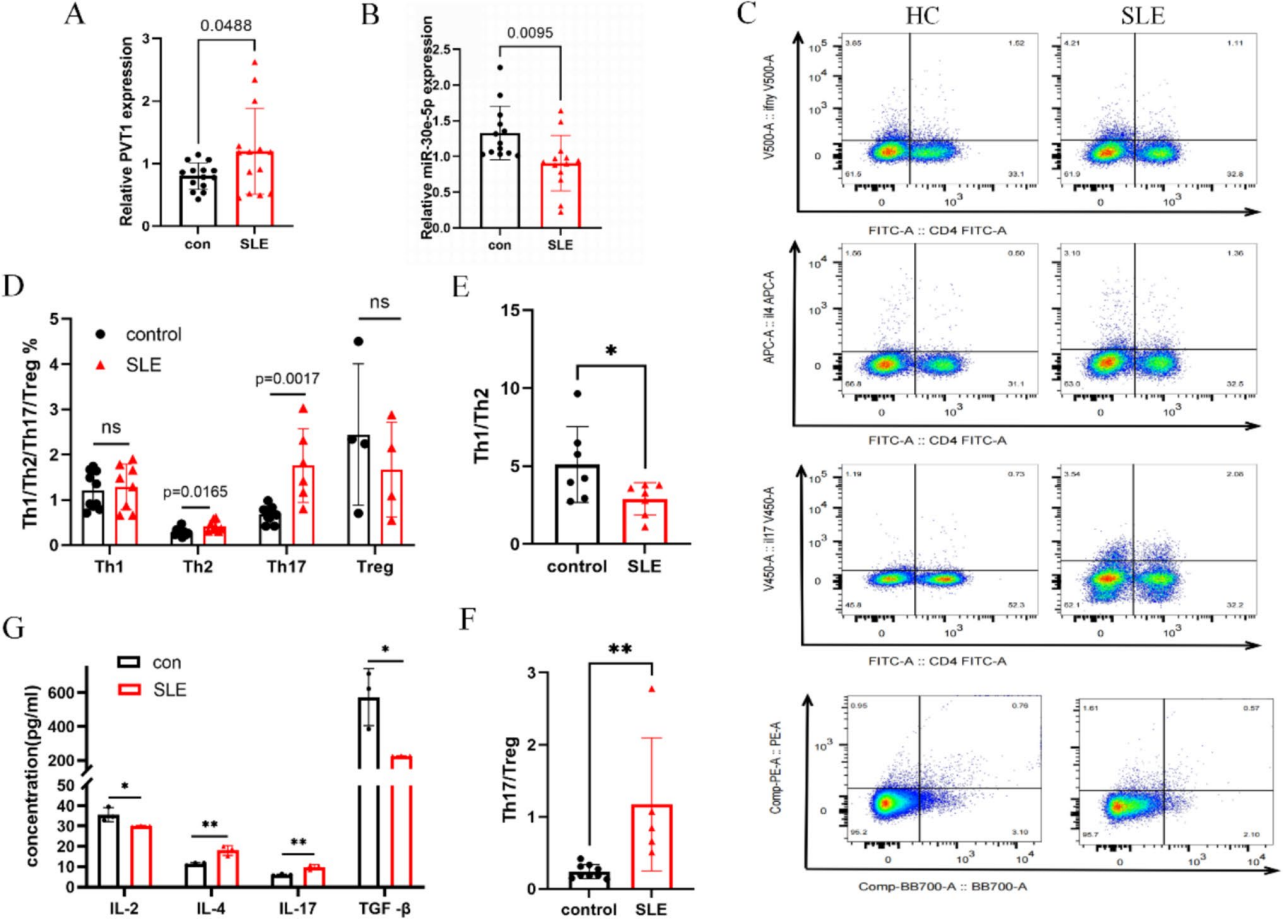
Increased lncRNA PVT1 expression and CD4^+^ T cell subset dysregulation in SLE. **B** lncRNA PVT1 (**A**) and miRNA-30e-5p (**B**) expression in peripheral blood from SLE patients vs HCs. **C** Representative dot plots of flow cytometry plots: IFN-γ/IL-4/IL-17 expression in stimulated CD4^+^ Th cells and CD25/Foxp3 co-expression. **D** Quantification of Th1 (CD4^+^IFN-γ^+^), Th2 (CD4^+^IL-4^+^), Th17 (CD4^+^IL-17 A^+^), and Treg (CD4^+^CD25^+^Foxp3^+^) subsets. **E**, **F** Th1/Th2 and Th17/Treg ratios. **G** Serum cytokine concentrations (IL-2, IL-4, IL-17, TGF-β). Data expressed as mean ± SEM. *P < 0.05; **P < 0.01; ***P < 0.001, ***P < 0.0001; ns, not significant

Quantitative RT-PCR revealed significantly elevated lncRNA PVT1 (*p* = 0.0488) and reduced miR-30e-5p (*p* = 0.0095) in SLE patients versus HCs (Fig. 1A, B). Flow cytometry demonstrated increased proportions of Th2 (*p* = 0.0165) and Th17 (*p* = 0.0017) cells, alongside decreased Th1 and Treg frequencies in SLE patients (Fig. 1C, D). Consequently, Th1/Th2 and Th17/Treg ratios were significantly imbalanced (*p* < 0.05; Fig. 1E–F), aligning with known CD4^+^ T cell dysregulation in SLE [3].

ELISA confirmed cytokine dysregulation: SLE patients exhibited elevated IL-4 (*p* = 0.0099) and IL-17 (*p* = 0.0069), but reduced IL-2 (*p* = 0.0464) and TGF-β (*p* = 0.0237), consistent with Th2/Th17 hyperactivity and impaired Th1/Treg function (Fig. 1G).

These findings provide additional confirmation of the presence of CD4 + T cell imbalance in the peripheral blood of SLE patients. Furthermore, we speculate that lncRNA PVT1 may contribute to the pathogenesis of SLE by influencing the CD4 + T cell imbalance.

### LncRNA PVT1 exacerbates the lupus phenotype in MRL/lpr mice

To systematically investigate the role of lncRNA PVT1 in SLE pathogenesis, CD4^+^ T cell dysregulation, and its interaction with miR-30e-5p, we generated lentiviral constructs for *PVT1* knockdown and overexpression in 40 female MRL-Fas(lpr) mice. Forty lupus mice were randomly assigned to one of four groups, each consisting of 10 mice: the blank control group (SLE), the SLE + lenti-Ctrl group (SLE + lenti-Ctrl), the SLE + lenti-si-*Pvt1* group (SLE + si-*Pvt1*), and the SLE + Lenti-*Pvt1* group (SLE + lenti-*Pvt1*).

**Fig. 2:**
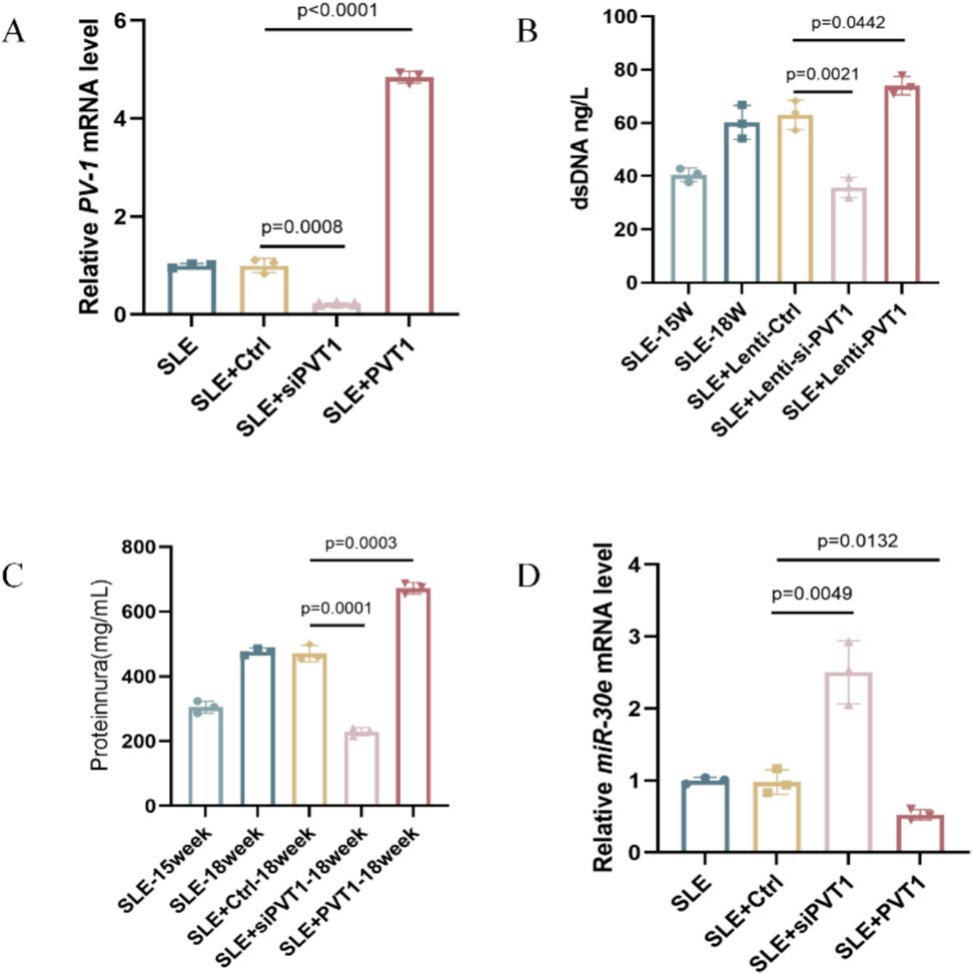
*PVT1* modulation in MRL/lpr mice. **A** *PVT1* expression at week 18. **B** Anti-dsDNA concentration. **C** Urinary protein levels. **D** *MiR-30e-5p* expression at week 18. Groups: SLE (untreated, 15 weeks and 18 weeks), SLE + lenti-Ctrl (empty vector, 18 W), SLE + lenti-si-*Pvt1* (*Pvt1* knockdown, 18 W), SLE + lenti-*Pvt1* (*Pvt1* overexpression, 18 W). Data expressed as mean ± SEM (*n* = 3)

Quantitative RT-PCR confirmed successful *Pvt1* modulation (Fig. 2A). Compared to the SLE + lenti-Ctrl group, *Pvt1* mRNA levels were reduced in the SLE + si-*Pvt1* cohort (*p* = 0.0008, one-way ANOVA with Tukey’s test) and elevated in the SLE + lenti-*Pvt1* group (*p* < 0.0001).

*Pvt1* perturbation directly correlated with SLE biomarker severity (Fig. 2B, C). Anti-dsDNA antibody concentrations decreased in SLE + si-*Pvt1* mice (*p* = 0.0021) and increased in SLE + lenti-*Pvt1* mice (*p* = 0.0442) versus SLE + lenti-Ctrl. Urinary protein levels mirrored this trend, with SLE + si-*Pvt1* mice exhibiting a reduction (*p* = 0.0001) and SLE + lenti-*Pvt1* mice an increase (*p* = 0.0003).

Notably, *Pvt1* and *miR-30e-5p* exhibited reciprocal expression patterns (Fig. 2D). *miR-30e-5p* levels rose by 2.1-fold in SLE + si-*Pvt1* mice (*p* = 0.0049) but declined by 45% in SLE + lenti-*Pvt1* mice (*p* = 0.0132), supporting a competitive endogenous RNA (ceRNA) interaction wherein *Pvt1* sequesters *miR-30e-5p* to derepress downstream targets.

### *LncRNA PVT1 contributes to CD4*^+^*T cell imbalance in MRL/lpr mice*

**Fig. 3:**
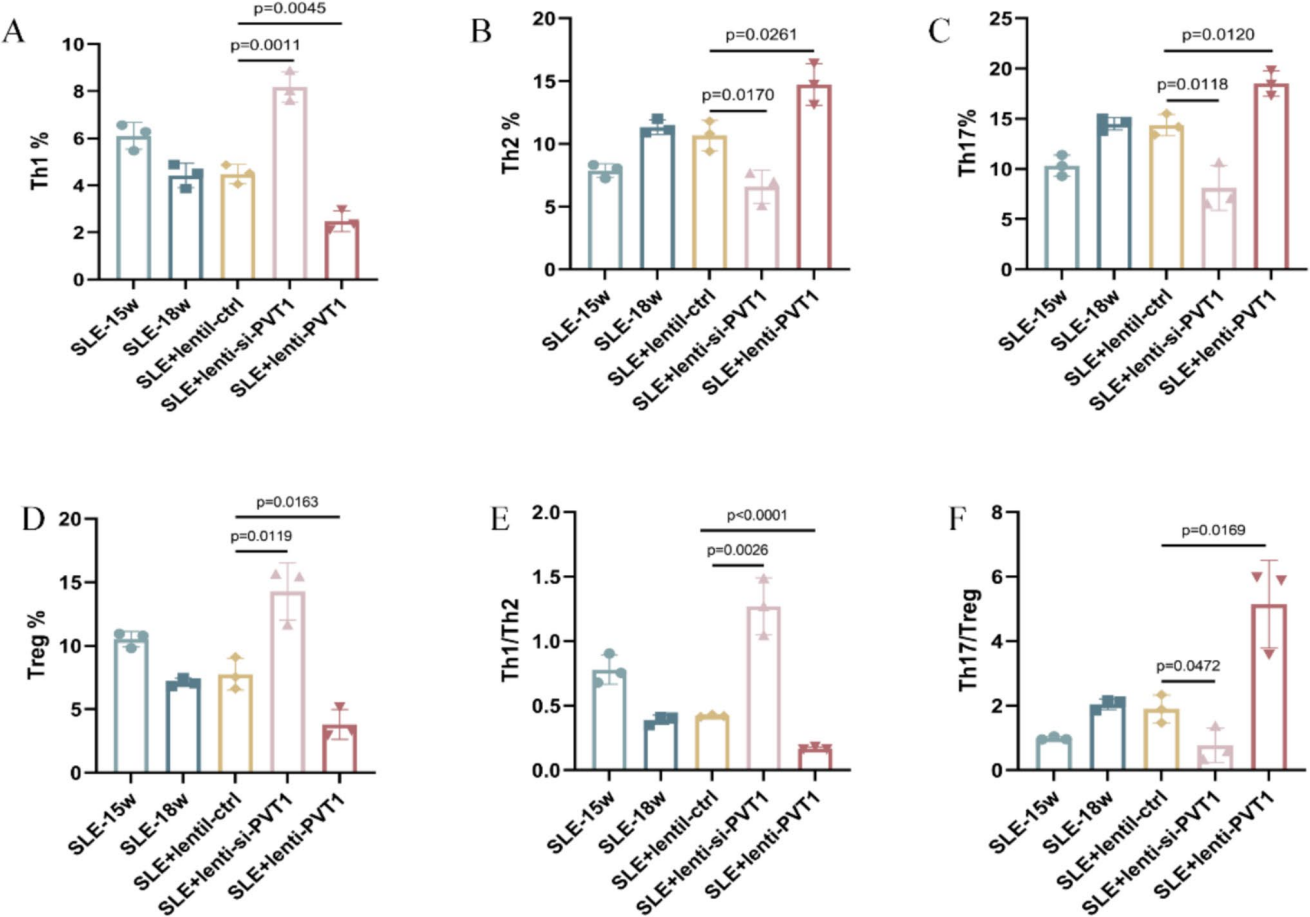
*Pvt1* regulates CD4 + T cell subset balance in MRL/lpr mice. **A**–**D** Circulating Th1 (**A**), Th2 (**B**), Th17 (**C**), and Treg (**D**) frequencies. **E**–**F** Th1/Th2 (**E**) and Th17/Treg (**F**) ratios. Groups: SLE (untreated, 15 weeks and 18 weeks), SLE + lenti-Ctrl (empty vector, 18 W), SLE + lenti-si-*Pvt1* (*Pvt1* knockdown, 18 W), SLE + lenti-*Pvt1* (*Pvt1* overexpression, 18 W). Data expressed as mean ± SEM (*n* = 3)

Flow cytometric analysis of peripheral blood CD4^+^ T cell subsets revealed robust modulation by *Pvt1* (Fig. 3A–D, Fig. S2–S5). Compared to the Lenti-Ctrl group, *Pvt1* knockdown (SLE + si-*Pvt1*) increased Th1 (CD4^+^IFN-γ^+^) cell frequency (*p* = 0.0011, one-way ANOVA with Tukey’s test) and Treg (CD4^+^CD25^+^Foxp3^+^) cells (*p* = 0.0119), while reducing Th2 (CD4^+^IL-4^+^) (*p* = 0.0017) and Th17 (CD4^+^IL-17 A^+^) (*p* = 0.0118) subsets, respectively. Conversely, *Pvt1* overexpression (SLE + lenti-*Pvt1*) decreased Th1 (*p* = 0.0045) and Treg (*p* = 0.0163) frequencies, respectively, but elevated Th2 (*p* = 0.0261) and Th17 (*p* = 0.0120) proportions.

Th1/Th2 and Th17/Treg ratios exhibited reciprocal trends (Fig. 3E–F). The Th1/Th2 ratio increased by 3.1-fold in SLE + si-*Pvt1* mice (*p* < 0.0026) but decreased by 67% in SLE + Lenti-*Pvt1* mice (*p* < 0.0001). Similarly, the Th17/Treg ratio declined by 72% with *Pvt1* knockdown (*p* = 0.0472) and rose by 2.4-fold with overexpression (*p* = 0.0169).

These data establish a causal relationship between *Pvt1* expression and CD4^+^ T cell polarization. *Pvt1* suppression skews immunity toward Th1/Treg dominance (pro-inflammatory resolution), while its overexpression exacerbates Th2/Th17 bias (pro-inflammatory amplification), directly disrupting CD4^+^ T cell homeostasis in lupus.

### *LncRNA PVT1 modulates CD4*^+^*T cell polarization through cytokine and transcriptional regulation*

To mechanistically link CD4^+^ T cell subset alterations to *Pvt1* activity, we quantified serum cytokines and lineage-specific transcription factors across experimental cohorts.

**Fig. 4:**
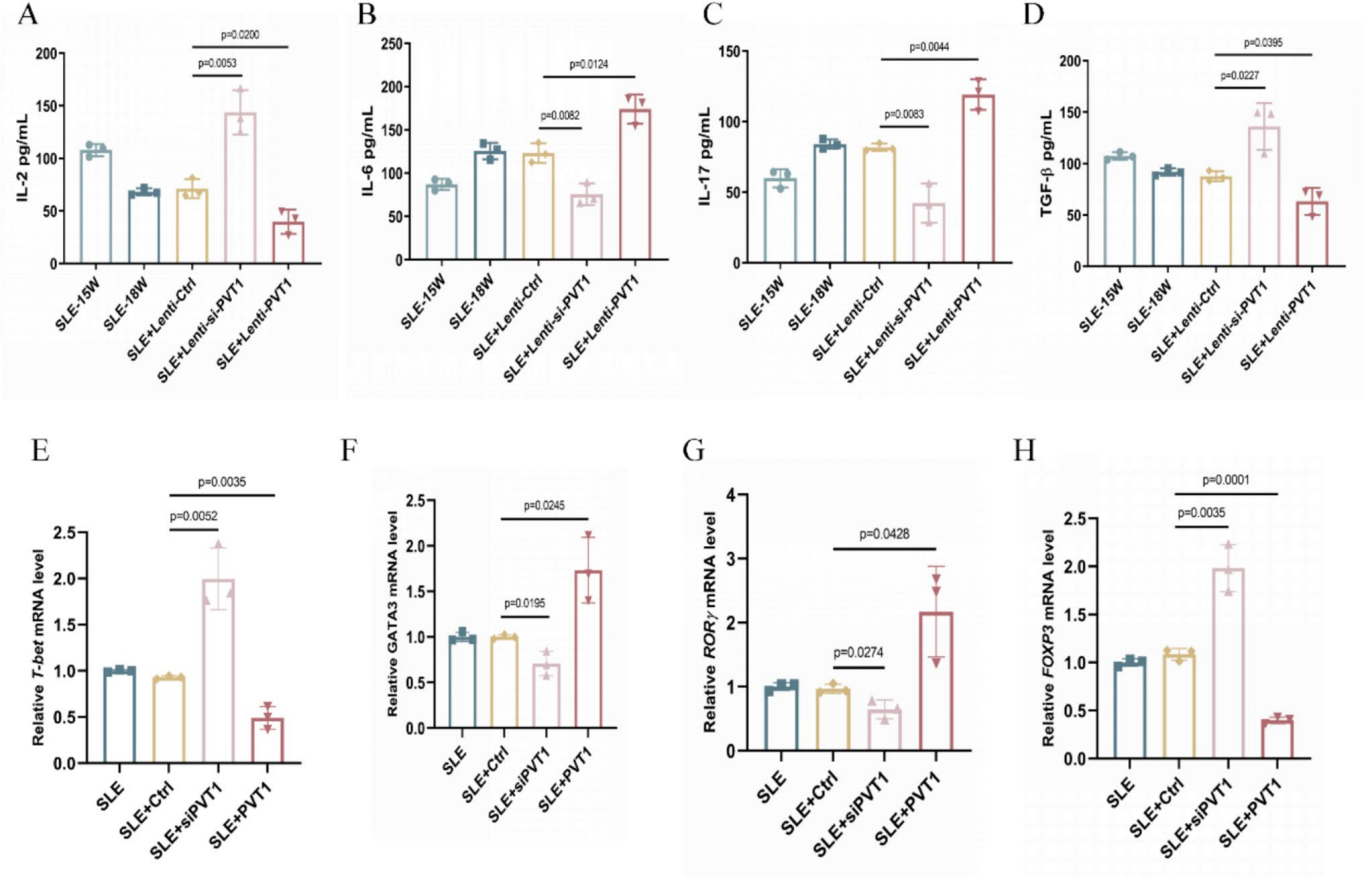
*Pvt1* modulates CD4.^+^ T cell-associated cytokines and transcription factors in MRL/lpr mice. **A**–**D** Cytokine concentrations: IL-2 (**A**), IL-6 (**B**), IL-17 (**C**), TGF-β (**D**). (E–H) mRNA levels of *T-bet* (**E**), *GATA3* (**F**), *RORγt* (**G**), and *Foxp3* (**H**). Group: SLE (untreated, 15 weeks and 18 weeks), SLE + lenti-Ctrl (empty vector, 18 W), SLE + lenti-si-*Pvt1* (*Pvt1* knockdown, 18 W), SLE + lenti-*Pvt1* (*Pvt1* overexpression, 18 W). Data expressed as mean ± SEM (*n* = 3)

*Pvt1* knockdown (SLE + si-*Pvt1*) significantly elevated levels of IL-2 (*p* = 0.0053) and TGF-β (*p* = 0.0227), while displaying significantly reduced levels of IL-6 (*p* = 0.0082) and IL-17 (*p* = 0.0083) compared to the SLE + lenti-Ctrl group (Fig. 4A–D). Conversely, *Pvt1* overexpression (SLE + lenti-*Pvt1*) showed significantly decreased concentrations of IL-2 (*p* = 0.0200) and TGF-β (*p* = 0.0395), along with significantly increased concentrations of IL-6 (*p* = 0.0124) and IL-17 (*p* = 0.0044) (Fig. 4A–D). Furthermore, we observed a consistent correlation between the trends of IL-2 and Th1; IL-6 and Th2; IL-17 and Th17; and TGF-β and Treg.

The lineage-determining transcription factors T-bet [26], GATA binding protein 3 (GATA3) [27], and retinoic acid-related orphan receptor gamma t (RORγt) [28] are critical for Th1, Th2, and Th17 cell differentiation and lineage maintenance, respectively. Regulatory CD4^+^ T cells (Treg) that express the lineage-specific transcription factor Forkhead box P3 (FoxP3) are essential for maintaining peripheral self-tolerance, thereby preventing and managing inflammation and autoimmunity throughout an individual’s lifespan [29].

At the transcriptional level, SLE + si-*Pvt1* upregulated Th1 master regulator *T-bet* (*p* = 0.0052) and Treg-specific *Foxp3* (*p* = 0.0035), while suppressing Th2-associated *GATA3* (*p* = 0.0195) and Th17-driving *RORγt* (*p* = 0.0274). SLE + lenti-*Pvt1* exerted opposite effects: *T-bet* (*p* = 0.0035) and *Foxp3* (*p* = 0.0001) were downregulated, whereas *GATA3* (*p* = 0.0245) and *RORγt* (*p* = 0.0428) were amplified (Fig. 4E–H).

This bidirectional regulation confirms *Pvt1* as a rheostat controlling CD4^+^ T cell lineage commitment. Through coordinated suppression of Th1/Treg programs (via IL-2↓/TGF-β↓, *T-bet*↓/*Foxp3*↓) and potentiation of Th2/Th17 pathways (via IL-6↑/IL-17↑, *GATA3*↑/*RORγt*↑), P*vt*1 overexpression establishes a pro-inflammatory milieu characteristic of active SLE.

## Discussion

SLE patients exhibit immune system activation characterized by abnormal activation of immune cells and breakdown of immune tolerance to self-antigens. T cells contribute significantly to the pathological development of SLE by promoting B cell responses and infiltrating target tissues, leading to tissue damage. In this study, we analyzed the gene expression of peripheral blood lncRNA PVT1 and miRNA-30e-5p in reproductive-age female SLE patients, revealing a specific high expression of lncRNA PVT1 in this cohort. Examination of CD4^+^ T cell subsets in peripheral blood revealed an imbalance of Th1/Th2 and Th17/Treg in SLE patients. Through the construction of SLE + si-*Pvt1* and SLE + lenti-*Pvt1* MRL/lpr mice, we additionally observed an inverse correlation between the expression patterns of lncRNA PVT1 and miRNA-30e-5p, along with the impact of lncRNA PVT1 on the phenotype of MRL/lpr mice and the imbalance of Th1/Th2 and Th17/Treg. Our findings indicate a specific upregulation of lncRNA PVT1 expression in female SLE patients, the influence of targeting lncRNA PVT1 on Th1/Th2 and Th17/Treg homeostasis, and the potential significance of lncRNA PVT1/miRNA-30e-5p in modulating immune responses in SLE through a ceRNA mechanism.

The reduced IL-2 and TGF-β levels in SLE patients (Fig. 1G) reflect impaired Th1 and Treg functionality, as IL-2 is essential for Th1 differentiation and Treg maintenance, while TGF-β drives Treg development and suppresses Th17 polarization. Conversely, elevated IL-6 and IL-17 (Fig. 1G) signify Th2/Th17 hyperactivity, promoting autoantibody production and tissue inflammation. In MRL/lpr mice, PVT1 knockdown restored IL-2 and TGF-β levels (Fig. 4A, D), correlating with increased Th1/Treg frequencies (Fig. 3A, D), whereas PVT1 overexpression exacerbated IL-6/IL-17 dominance (Fig. 4B, C), aligning with Th2/Th17 expansion (Fig. 3B, C). These cytokine shifts mirror SLE’s hallmark pathology: Th1/Treg deficiency permits loss of self-tolerance, while Th2/Th17 skewing drives chronic inflammation and organ damage. Thus, PVT1-mediated cytokine dysregulation directly links CD4^+^ T cell subset imbalance to clinical manifestations like autoantibody production (anti-dsDNA) and renal injury (proteinuria) (Fig. 2B, C).

The lncRNAs are transcribed by the RNA Pol ll, but unlike mRNAs, they do not serve templates for protein synthesis. Their function is associated with the control of gene expression at different levels, such as its association with chromatin for transcription controlling, IncRNA-mRNA interaction for mRNA translation controlling, and the IncRNA-protein interaction. Therefore, IncRNAs can interact with all types ofi nformational molecules in mammalian cells whose function is associated with the control of gene expression flow, chromatin remodeling, and epigenetic regulation [30] [31]. CD4^+^ T cells differentiate into distinct subpopulations in response to immune challenges, regulated by lineage-specific cytokines and transcription factors. Nevertheless, the differentiation mechanisms are complex and extend beyond these factors.

Th2 cells are responsible for chronic respiratory inflammation in asthma [32]. During ozone-induced asthma exacerbation, PVT1 activates the phosphatidylinositol 3-kinase (PI3 K)/AKT/mammalian target of rapamycin (mTOR) pathway by inhibiting the miR-15a-5p pathway, resulting in a reduced Th1/Th2 ratio that facilitates asthma progression. Elevated levels of IL-4, IL-10, and GATA3 proteins were observed, whereas IFN-γ, IL-2, and T-bet levels were decreased [33]. In allergic rhinitis, PVT1 exacerbates inflammation, resulting in an imbalance between Th1 and Th2 cells. Increased PVT1 expression correlated negatively with Th1 cells (*p* = 0.028) and showed a decreasing trend with IFN-γ (*p* = 0.065), while positively correlating with Th2 cells (*p* = 0.012) and IL-10 (*p* = 0.021) [34]. Our study further demonstrated that SLE + si-*Pvt1* significantly increased the frequencies of Th1 and Treg cells while decreasing Th2 and Th17 cells in MPL/lpr mice. This indicates that PVT1 supports the development of both Th1 and Th2 cells in various clinical contexts, potentially due to the specific expression of lncRNAs in different cells or the structural characteristics of PVT1.

Numerous studies suggest that aberrant expression of lncRNA PVT1 may contribute to the pathogenesis of autoimmune diseases. Elevated levels of PVT1 in osteoarthritis have been shown to promote chondrocyte apoptosis [35]. In MRL/lpr lupus mice, increased PVT1 expression is hypothesized to enhance autoantibody production by accelerating somatic cell apoptosis, resulting in elevated nuclear antigens in vivo. Whereas in rheumatoid arthritis, PVT1 knockdown suppressed IL-1β and IL-6 expression, consistent with our findings of significantly reduced IL-6 expression in the SLE + si-*Pvt1* group, while also activating apoptosis in fibroblast-like synoviocytes [36]. These contrasting results may stem from lupus pathology, which is characterized by both heightened apoptosis and inadequate clearance of apoptotic debris [37].

PVT1 has been shown to bind miRNAs through its miRNA-binding domain (MBD), influencing their expression and function, which in turn affects target gene expression. lncRNA PVT1 contributes to the process of CD4^+^ T cell differentiation through its interaction with miRNAs. Dysregulation of miRNAs has been implicated in aberrant immune responses and organ damage in SLE patients. PVT1 functions as a ceRNA and competitively binds to miR-200 family members, interrupting their function [38]. Circulating miR-200 family members serve as biomarkers for SLE and may modulate the Th17/Treg ratio, which plays a pivotal role in SLE pathophysiology and disease activity [39]. Overexpression of PVT1 in Tregs suppresses miR-146a, leading to TRAF6 upregulation [40]. Conversely, the downregulation of miR-146a in SLE promotes type I interferon signaling, enhancing autoimmunity [41]. Kim et al. investigated circulating miRNAs associated with SLE susceptibility in a Korean population and revealed significant upregulation of hsa-miR-30e-5p, hsa-miR-92a-3p, and hsa-miR-223-3p in the plasma of SLE patients compared to healthy controls [42]. Cheng et al. demonstrated that RvD1 mitigates SLE progression by upregulating Treg and downregulating Th17 cells via miR-30e-5p [43].

Recent investigations revealed significantly reduced serum PVT1 levels in SLE patients compared to healthy controls. Notably, diminished serum PVT1 levels correlated with oral ulceration and neurological manifestations related to photosensitivity. Additionally, serum PVT1 negatively correlated with age (*r* = − 0.52, *p* < 0.0001) and erythrocyte sedimentation rate (ESR) (*r* = − 0.29, *p* = 0.011) among SLE patients. However, no correlation was observed between PVT1 and urinary protein levels or anti-dsDNA antibody levels in SLE patients [44]. Our study focused on plasma PVT1 levels, while previous studies investigated serum levels. Considering the presence of CD4^+^ T cells in plasma, we assert that our results are more aligned with our research objectives. Additionally, to account for gender heterogeneity in SLE [45], particularly its higher prevalence among women of reproductive age, we recruited age-matched female SLE patients and healthy controls to minimize within-group variability.

This study positions PVT1 as a master regulator of CD4^+^ T cell homeostasis and a promising therapeutic target. Pharmacological inhibition of PVT1 (e.g., antisense oligonucleotides or CRISPR-based silencing) could restore miR-30e-5p activity, rebalancing Th1/Th2 and Th17/Treg ratios. Such strategies may complement existing therapies targeting cytokine pathways (e.g., anti-IL-17 biologics) by addressing upstream epigenetic drivers of immune dysregulation. Furthermore, circulating PVT1 levels could serve as a dynamic biomarker for monitoring disease activity or treatment response. The sample size, though sufficient for initial insights, warrants validation in larger cohorts to account for SLE heterogeneity. Future studies should prioritize three directions: (1)validating direct PVT1-miR-30e-5p interactions through RIP-seq and luciferase reporter assays; (2) investigating tissue-specific effects of PVT1 modulation in target organs (e.g., kidneys, skin); and (3) exploring synergistic effects of PVT1 inhibition with conventional immunosuppressants in preclinical models.

Our findings establish lncRNA PVT1 as a pivotal regulator of CD4^+^ T cell imbalance in SLE, mediated via the miR-30e-5p-dependent ceRNA network. By skewing T cell differentiation toward pro-inflammatory Th2/Th17 subsets and impairing Th1/Treg function, PVT1 amplifies cytokine-driven tissue damage. Targeting PVT1 represents a promising strategy to restore immune homeostasis, with potential for clinical translation. Future work should address mechanistic nuances and therapeutic optimization, advancing our understanding of lncRNA-driven autoimmunity.

## Data Availability

All data that supports the findings of this study are available from the corresponding author upon reasonable request.
